# Occupations at high risk for malaria in Zanzibar: a case–control study, may–august 2023

**DOI:** 10.1186/s12936-025-05517-0

**Published:** 2025-08-20

**Authors:** Geofrey Makenga, Sarah Gallalee, Humphrey Mkali, Mwinyi Issa Khamis, Abdulhamid Ramadhan, Mohamed Haji Ali, Wahida S. Hassan, Juma Hassan, Stella Makwaruzi, Saidi Mgata, Michael Gulaka, Nicodem James Govella, Fabrizio Molteni, Chonge Kitojo, Erik Reaves, Sarah-Blythe Ballard, Naomi Serbantez, Albert Ikonje, Marguerite Afenu, Sigisbert Mkude, Jennifer L. Smith, Safia Mohamed, Shija J. Shija, Cara Smith Gueye, Roly Gosling

**Affiliations:** 1 PMI Dhibiti (Control) Malaria Project, Population Services International, Dar es Salaam, United Republic of Tanzania; 2 Malaria Elimination Initiative, University of California, San Francisco, USA; 3 Zanzibar Malaria Elimination Programme (ZAMEP), Ministry of Health, MjiniMagharibi, Zanzibar, United Republic of Tanzania; 4 Ifakara Health Institute, Dar es Salaam, United Republic of Tanzania; 5 Swiss Tropical Public Health, Dar es Salaam, United Republic of Tanzania; 6 U.S. President’s Malaria Initiative, US Agency for International Development, Dar es Salaam, United Republic of Tanzania; 7 U.S. President’s Malaria Initiative, US Centers for Disease Control and Prevention, Dar es Salaam, United Republic of Tanzania; 8 Population Services International, Washington, DC USA

## Abstract

**Background:**

In malaria elimination settings, cases tend to cluster geographically and occur among certain subpopulations. Clustering is often related to specific factors such as occupation or mobility, which increase an individual’s risk for malaria infection.

**Methods:**

A case–control study was conducted to identify malaria high-risk populations (HRPs) in Zanzibar. Patients presenting with symptoms of malaria at selected facilities were recruited from historically high burden areas in two urban districts (Mjini and Magharibi B) and two rural districts (Kati and Micheweni). Between May and August 2023, the study recruited 197 cases and 557 controls frequency matched by age group and sex. Logistic regression was used to explore associations between risk factors and the epidemiological outcome of local malaria infection, classified as confirmed malaria cases with no travel outside Zanzibar in the prior 3 weeks.

**Results:**

In urban districts, night watchmen/police (odds ratio [OR] 5.3, 95% confidence interval [CI]: 2.7–10.6, *p* < 0.001), construction workers (OR 3.0, 95% CI 1.8–5.0 *p* = 0.007), and farmers (OR 1.6, 95% CI 1.1–2.2, *p* = 0.01) were found to have higher odds of malaria infection compared to those not working in those professions. Other high-risk behaviours in urban districts included night-time activities (OR 2.8, 95% CI 1.8–4.3, *p* < 0.001), meals taken outside (OR 2.0, 95% CI 1.1–3.4, *p* = 0.01), and recent travel within Zanzibar (OR 3.3, 95% CI 1.5–7.1, *p* = 0.002). In rural districts, outdoor night-time activities (OR 3.8, 95% CI 1.5–9.9,* p* = 0.006) and taking meals outside (OR 2.7, 95% CI 1.1–6.6, *p* = 0.03) were risk factors for malaria; however, no higher risk occupational groups were identified. Overall, there was a trend towards net use being protective against malaria, but this association only reached statistical significance in rural districts (*p* = 0.015).

**Conclusion:**

Tailored interventions targeting specific occupational groups could be an effective strategy to reduce malaria in urban areas in Zanzibar.

## Background

Zanzibar has achieved a tremendous decline in malaria cases in recent years; cases declined 62% from 11,613 in 2019 to 4389 in 2022 [1, 2]. Successes in achieving this decline are mainly attributed to repeated and sustained programme-led and partner-supported vector control, case management, and surveillance interventions [3–5]. However, malaria cases surged in Zanzibar in 2023, increasing to 18,935 cases (of which 18,283 (97%) cases came from Unguja Island alone). Zanzibar is considered a malaria pre-elimination setting, with an overall incidence of 2 per 1000 population in 2022—specifically, 1 per 1000 in Pemba Island and 2 per 1000 in Unguja Island. This increased markedly in 2023 to 13 per 1000, a year classified as a surge year, although Pemba Island maintained a low incidence of 1 per 1000. While malaria importation from mainland Tanzania continuous to pose a challenge to elimination efforts, local transmission also persists. In Unguja island, 19% of cases were classified as local in 2022, rising sharply to 78% in 2023 [2, 6]. *Plasmodium falciparum* is the predominant species of malaria in Zanzibar; *Plasmodium vivax* is rare [2, 6].

In elimination settings, malaria increasingly clusters in certain geographic areas and among subpopulations at higher risk of infection [8]. Malaria “high-risk populations” (HRPs) are groups of people who share socio-demographic, geographic and/or behavioral characteristics that place them at higher risk of infection, such as low access or utilization of health services and interventions, or activities associated with increased exposure to malaria vectors such as *Anopheles* mosquitoes [8]. Generating a more detailed understanding of the priority malaria HRPs in specific geographies, associated high-risk activities and potential approaches to access them for diagnosis, treatment, surveillance and response, and prevention interventions will be essential to Zanzibar’s efforts to reduce local transmission of malaria in alignment with the current malaria strategic plan for the islands.

While the Zanzibar Malaria Elimination Programme (ZAMEP) routinely collects information on potential risk factors of occupation or travel, which helps to characterize HRPs, the data tend to be broad (e.g., “business”) and do not capture more detailed behaviour or specific occupation groups. More detail is needed to describe risk among specific age groups, sexes, occupations, and behaviours in order to understand the types of interventions and methods of delivering interventions that would be useful [9].

Further, identifying and characterizing HRP groups in priority transmission areas that are likely to have a high degree of local, not imported, transmission in Zanzibar, is necessary to target and tailor malaria responses. Recent studies have demonstrated the utility of using adapted case–control study designs for generating evidence and interpreting data to inform intervention design and identify and characterize HRPs [10–13]. This study uses a case–control design to identify risk factors for local malaria in Zanzibar.

## Methods

### Study area

**Fig. 1:**
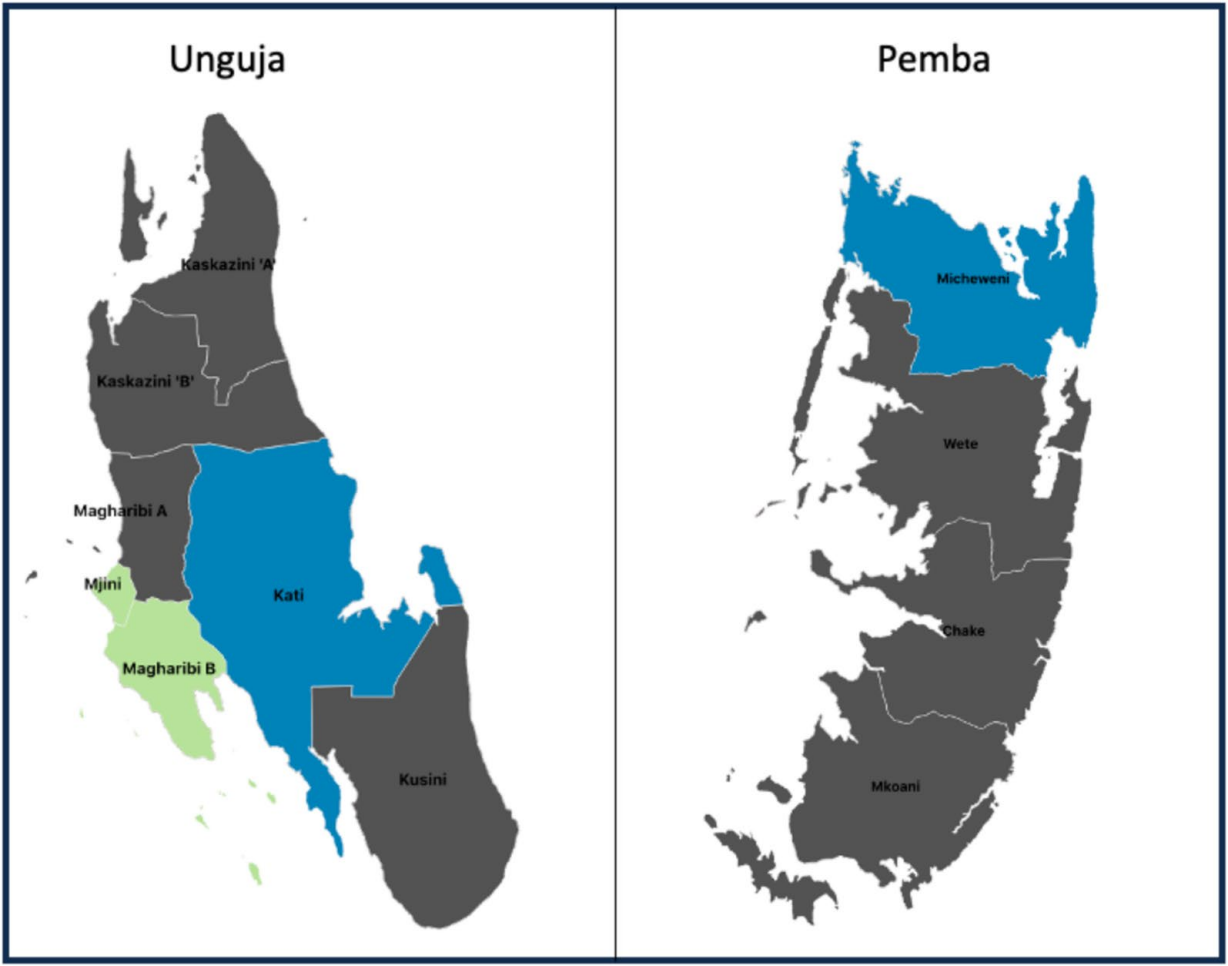
Study districts. Districts shown in green are considered urban, districts shown in blue are considered rural

Zanzibar is an archipelago located in the Indian Ocean off the northeast coast of Tanzania; it consists of many small islands and two main islands of Unguja and Pemba (Fig. 1). The population of Zanzibar is an estimated 1.8 million people [14]. Zanzibar is semi-autonomous and has its own Ministry of Health encompassing ZAMEP, which implements malaria elimination activities [3]. Malaria transmission in Zanzibar historically aligns with the bimodal rainfall seasons: one from March to June and the second from October to November [3, 15].

This study was conducted in thirty-seven higher-burden health facilities across four districts of Zanzibar that had the highest number of local cases in 2021 and 2022 during the proposed study months (May–October). The districts were two urban districts (Magharibi B and Mjini) and two rural districts (Kati and Micheweni). Michiweni district is on the Island of Pemba and the other three districts are on the island of Unguja (Fig. 1). The urban districts are characterized by higher density of population and more developed infrastructure; the rural districts are characterized by lower density of population and are largely farmland or undeveloped land.

### Study design

A prospective case–control study on risk factors for local malaria was conducted from May through August 2023 to compare the characteristics of people with malaria (cases) to the characteristics of those without malaria (controls), nested within the existing surveillance system. Local malaria was defined as any malaria case with no travel history outside of Zanzibar in the previous 30 days. The study period selected was an anticipated high transmission season based on historical data. The UCSF-developed Malaria Elimination Guide to Targeted Surveillance and Response in High-Risk Populations (HRP Guide) was used to inform the study design, as used elsewhere to identify high risk groups [16].

The study population included patients presenting to study health facilities with suspected locally-acquired malaria to receive routine malaria testing services; patients were eligible to participate as a case if they were confirmed positive by RDT or microscopy, in alignment with national guidelines, and were eligible to participate as a control if they tested negative. All participants were required to be 15 years or older and be willing and available to participate in the study; exclusion criteria included travel outside of Zanzibar in the past 3 weeks and testing positive for malaria in the past month. Controls were frequency matched by age group (i.e. 15–29, 30–44, 45 +) and sex and recruited in a 1:3 case to control ratio. The age categories were selected to match age groups used in routine programmatic work in Zanzibar. Controls were recruited in fixed numbers each month; targets were set for each month and a recruitment tracker was used at each health facility based on the expected number of monthly cases at each facility [16]. Sample size calculations were estimated separately for rural and urban areas and determined a minimum of 130 cases and 390 controls were needed overall to provide 80% power to demonstrate a minimum odds ratio of 3.4 with an alpha of 0.05 [16].

### Data collection

The Coconut Platform hosts routine data collection for malaria case notification and investigation in Zanzibar [17]. Health facility workers routinely use this system for malaria case reporting. In this study, the data collection survey was set up as a separate component within the Coconut Platform to enable ease of use by health facility staff. These electronic surveys were conducted on tablets connected to the Coconut Platform. Data were stored within the Coconut system in a database separate from the routine surveillance database.

All patients at participating health facilities received a malaria test based on signs and symptoms of uncomplicated malaria in line with national guidelines and were screened by trained health facility staff for eligibility to participate in the study. All eligible cases were individually administered informed consent as well as a subset of eligible controls selected based on matching variables. Consenting participants were interviewed by a health facility staff member on the same day using the electronic study questionnaire in Swahili (attached as supplementary document). No population or sub-population was sought out specifically during this study, all individuals enrolled as a case or control sought care at a participating health facility and were enrolled if criteria were met.

### Data processing

Data management, cleaning, and analysis were conducted using Stata and R. Each occupation was included as a separate binary variable (e.g., fisherman: yes, vs. no) to allow for people who reported multiple occupations. Categories within variables that were similar were combined to avoid cells smaller than five (e.g., “construction/factory/carpenter”). The “watchmen/police” category was collected as one group; therefore, this category could not be separated into two individual groups for sub-analysis. The occupation category of “other” included occupations with too few observations to be standalone groups; examples include hotel worker, barber, tailor and mechanic. The category “outside activities at night” was defined as individuals who reported doing activities outside at night during the past month. “Outside meals” includes individuals who reported taking meals outside in the past month. Recent travel includes those who reported traveling within Zanzibar in the past 3 weeks and staying overnight.

### Data analysis

Rural and urban districts were assessed in separate analyses using unconditional logistic regression to explore associations between risk factors and the outcome of local malaria infection [16, 18]. The study assessed the relationships between the main variables of interest (occupation, night-time activities, meals taken outside, prior night net use, and travel) and the outcome of locally-acquired malaria after adjusting for age group, sex, and district as fixed effects and clustered standard errors by health facility.

## Results

### General description

During May to August 2023, 197 cases and 557 controls were recruited across the four study districts, the majority in Magharibi B and Mjini. A wide variety of occupations were captured; Table 1 presents the primary occupations reported by participants. Employment in more than one occupation was reported by 175 participants (23%). Overall, more cases than controls reported spending time outside at night, taking meals outside, and traveling overnight within Zanzibar during the past 3 weeks. Cases were more frequently employed as night watchmen/police compared to controls.

**Table 1:** Overall study participant demographics

	Case	Control	Overall
	(N = 197)	(N = 557)	(N = 754)
District			
Kati	29 (14.7%)	74 (13.3%)	103 (13.7%)
Magharibi B	95 (48.2%)	284 (51.0%)	379 (50.3%)
Micheweni	8 (4.1%)	18 (3.2%)	26 (3.4%)
Mjini	65 (33.0%)	181 (32.5%)	246 (32.6%)
Sex			
Female	54 (27.4%)	167 (30.0%)	221 (29.3%)
Male	143 (72.6%)	390 (70.0%)	533 (70.7%)
Age Group (years)			
15–29	133 (67.5%)	360 (64.6%)	493 (65.4%)
30–44	43 (21.8%)	134 (24.1%)	177 (23.5%)
45 +	21 (10.7%)	63 (11.3%)	84 (11.1%)
Primary Occupation			
Construction/Factory/Carpenter	20 (10.2%)	39 (7.0%)	59 (7.8%)
Farmer/Animal Husbandry	26 (13.2%)	55 (9.9%)	81 (10.7%)
Fisherman	10 (5.1%)	26 (4.7%)	36 (4.8%)
Housewife	24 (12.2%)	61 (11.0%)	85 (11.3%)
Large scale whole sale	4 (2.0%)	9 (1.6%)	13 (1.7%)
Motorcyclist (Bodaboda)	5 (2.5%)	22 (3.9%)	27 (3.6%)
Other	18 (9.1%)	87 (15.6%)	105 (13.9%)
Other sales	5 (2.5%)	19 (3.4%)	24 (3.2%)
Small scale food vendor	12 (6.1%)	43 (7.7%)	55 (7.3%)
Small shop (kiosk)	8 (4.1%)	36 (6.5%)	44 (5.8%)
Student	19 (9.6%)	63 (11.3%)	82 (10.9%)
Unemployed	27 (13.7%)	84 (15.1%)	111 (14.7%)
Watchman/Police	19 (9.6%)	13 (2.3%)	32 (4.2%)
Outside activities at night			
No	110 (55.8%)	433 (77.7%)	543 (72.0%)
Yes	87 (44.2%)	124 (22.3%)	211 (28.0%)
Outside meals			
No	145 (73.6%)	476 (85.5%)	621 (82.4%)
Yes	52 (26.4%)	81 (14.5%)	133 (17.6%)
Prior night net use			
No	98 (49.7%)	234 (42.0%)	332 (44.0%)
Yes	99 (50.3%)	323 (58.0%)	422 (56.0%)
Recent travel			
No	149 (75.6%)	501 (89.9%)	650 (86.2%)
Yes	48 (24.4%)	56 (10.1%)	104 (13.8%)

### Urban districts

In the urban districts of Magharibi B and Mjini, there were 160 cases and 465 controls. Most urban participants were male (72%) and in the age group of 15–29 (66%). The most common occupations were construction/factory/carpenter, watchman/police, unemployed, and housewife. It was common to report more than one occupation (22%).

Occupations with high levels of outdoor activities were associated with increased risk of malaria. Table 2 presents odds ratios with 95% confidence intervals for potential risk factors for malaria in the urban districts, adjusting for sex, age group, and district as fixed effects and health facility as a cluster variable. Three occupations were strongly associated with malaria risk in this population. Participants who reported working as night watchmen/police had 5.34 times the odds of malaria (95% CI 2.69–10.58) compared to those who did not report working in this occupational category. The two other job categories with higher odds of malaria were construction/factory/carpenter (OR: 2.00, 95% CI 1.20–3.32) and farmer/animal husbandry (OR: 1.56, 95% CI 1.09–2.21). This association remained significant when the category of construction occupation was assessed individually (OR: 2.96, 95% CI 1.76–4.99, p < 0.001). Factory workers and carpenters were not large enough groups to assess individually. In the job category of “other”, individuals had 0.55 times the odds of malaria (95% CI 0.31–0.98).

**Table 2:** Urban districts: odds ratios for logistic regression

Occupation category	Cases	Controls	Odds Ratio (95% CI)	*P* value
	N = 160	N = 465		
Construction/Factory/Carpenter	22 (14%)	35 (8%)	2.00 (1.2–3.32)	0.007
Farmer/Animal Husbandry	19 (12%)	38 (8%)	1.56 (1.09–2.21)	0.014
Fisherman	10 (6%)	21 (5%)	1.40 (0.56–3.5)	0.467
Housewife	20 (12%)	51 (11%)	1.52 (0.77–2.99)	0.227
Large scale whole sale*	–	–	–	–
Motorcyclist (Boda-boda)	7 (4%)	25 (5%)	0.76 (0.36–1.6)	0.473
Other	17 (11%)	81 (17%)	0.55 (0.31–0.98)	0.042
Other sales	10 (6%)	34 (7%)	0.84 (0.55–1.28)	0.413
Small scale food vender	11 (7%)	42 (9%)	0.77 (0.45–1.34)	0.359
Small shop (kiosk)	6 (4%)	36 (8%)	0.44 (0.17–1.12)	0.086
Student	17 (11%)	55 (12%)	0.83 (0.52–1.32)	0.424
Unemployed	20 (12%)	73 (16%)	0.74 (0.46–1.18)	0.204
Watchman/Police	20 (12%)	13 (3%)	5.34 (2.69–10.58)	< 0.001
Outside activities at night	71 (44%)	106 (23%)	2.78 (1.8–4.3)	< 0.001
Outside meals	41 (26%)	68 (15%)	2.00 (1.18–3.39)	0.01
Prior night net use	83 (52%)	263 (57%)	0.84 (0.57–1.23)	0.374
Recent travel	41 (26%)	48 (10%)	3.31 (1.54–7.13)	0.002

Footnote: Each individual model adjusted for sex, age group, and district as fixed effects and health facility as a cluster variable. Occupations are included as separate binary variables each occupation category includes individuals who listed it as a primary or secondary occupation

*Denotes merged with other groups to avoid small cells

Outside activities at night in the past month (outside between 6 pm and 6am), taking meals outside in the past month, and traveling within Zanzibar in the last 3 weeks were all associated with malaria risk in the urban districts (Table 2). There was a trend towards a protective effect of net use the prior night that did not reach statistical significance in urban areas. Of those who reported engaging in night-time activities outside in the past month (28.3% of urban participants), only 48.6% reported sleeping under a net the prior night.

The three malaria high-risk occupation groups in urban areas are described in Table 3. Cases among all three of these groups (night watchmen/police, construction workers/factory/carpenter, and farmers) reported more recent travel within Zanzibar compared to controls (p < 0.05). Among night watchmen/police, 95% of cases reported night time activities compared to 46% of controls (p = 0.001).

**Table 3:** Characteristics of identified malaria high-risk occupation groups in urban districts

Night watchmen/police	Cases	Controls	*p*-value
	N = 20	N = 13	
Sex			–
Female	0 (0%)	1 (8%)	
Male	20 (100%)	12 (92%)	
Age Group			–
15–29 years	8 (40%)	7 (54%)	
30–44 years	9 (45%)	4 (31%)	
45 + years	3 (15%)	2 (15%)	
Outside activities at night	19 (95%)	6 (46%)	0.001
Outside meals	9 (45%)	2 (15%)	0.078
Prior night net use	10 (50%)	5 (38%)	0.52
Recent travel	10 (50%)	1 (8%)	0.012

Construction/factory/carpenter	Cases	Controls	*p*-value
	N = 22	N = 35	
Sex			–
Female	1 (5%)	2 (6%)	
Male	21 (95%)	33 (94%)	
Age Group			–
15–29 years	13 (59%)	19 (54%)	
30–44 years	5 (23%)	12 (34%)	
45 + years	4 (18%)	4 (11%)	
Outside activities at night	10 (45%)	8 (23%)	0.074
Outside meals	9 (41%)	8 (23%)	0.15
Prior night net use	12 (55%)	21 (60%)	0.68
Recent travel	8 (36%)	2 (6%)	0.003

Farmer/Animal Husbandry	Cases	Controls	*p*-value
	N = 19	N = 38	
Sex			–
Female	2 (11%)	5 (13%)	
Male	17 (89%)	33 (87%)	
Age Group			–
15–29 years	13 (68%)	22 (58%)	
30–44 years	3 (16%)	9 (24%)	
45 + years	3 (16%)	7 (18%)	
Outside activities at night	6 (32%)	8 (21%)	0.38
Outside meals	4 (21%)	5 (13%)	0.44
Prior night net use	9 (47%)	20 (53%)	0.71
Recent travel	4 (21%)	1 (3%)	0.02

*p*-values are not included for sex and age group as these were matching variables

### Rural districts

In the rural districts of Kati and Micheweni, there were 37 cases and 92 controls. Similar to the urban districts, the majority of rural participants were male (63%) and in the age group of 15–29 (61%). The most common occupation was farmer/animal husbandry. It was common to report more than one occupation (30%).

Table 4 presents odds ratios with 95% confidence intervals for potential risk factors for malaria in the rural districts. There were no salient occupational risk factors in the rural districts. Nighttime activities (OR: 3.81, 95% CI 1.47–9.91) and taking meals outside at night (OR: 2.72, 95% CI 1.11–6.64) were associated with higher odds of malaria compared to not participating in those activities. Recent travel within Zanzibar trended towards an association with risk for malaria but did not reach statistical significance. In the rural districts, prior night net use was associated with lower odds of malaria compared to not using a net (OR: 0.38, 95% CI 0.18–0.83). Of those who reported conducting night-time activities outside in the past month (26.4% of rural participants), 50.0% reported sleeping under a net the prior night.

**Table 4:** Rural districts: odds ratios for logistic regression

Occupation Category	Cases	Controls	Odds Ratio (95% CI)	*P* value
	N = 37	N = 92		
Construction/Factory/Carpenter*	–	–	–	–
Farmer/Animal Husbandry	11 (30%)	29 (32%)	0.86 (0.40–1.85)	0.692
Fisherman*	–	–	–	–
Housewife	8 (22%)	25 (27%)	0.66 (0.26–1.65)	0.377
Large scale whole sale*	–	–	–	–
Motorcyclist (Bodaboda)*	–	–	–	–
Other	10 (27%)	40 (43%)	0.43 (0.16–1.15)	0.093
Other sales	5 (14%)	9 (10%)	1.60 (0.59–4.33)	0.353
Small scale food vender*	–	–	–	–
Small shop (kiosk)*	–	–	–	–
Student*	–	–	–	–
Unemployed	7 (19%)	11 (12%)	1.74 (0.59–5.08)	0.314
Watchman/Police*	2 (5%)	2 (2%)		
Outside activities at night	16 (43%)	18 (19.6%)	3.81 (1.47–9.91)	0.006
Outside meals	11 (30%)	13 (14%)	2.72 (1.11–6.64)	0.028
Prior night net use	16 (43%)	60 (65%)	0.38 (0.18–0.83)	0.015
Recent travel	7 (19%)	8 (9%)	2.60 (0.83–8.04)	0.097

Footnote: Each individual model adjusted for sex, age group, and district as fixed effects and health facility as a cluster variable. Occupations are included as separate binary variables each occupation category includes individuals who listed it as a primary or secondary occupation

*Denotes merged with other groups to avoid small cells

## Discussion

In Zanzibar, imported infections from mainland Tanzania are typically studied with the assumption that there is limited local transmission in the archipelago [7, 19]. However, this study shows that there are high-risk groups in relation to local transmission who have occupational and behavioural exposures that might benefit from tailored interventions. This study also observed a lower proportion of female malaria cases, which was expected. Recent epidemiologic trends in Zanzibar show that malaria is primarily affecting males between 15 and 45 years old. Characterizing groups that have higher occupational risk and other risk factors for malaria allows for the malaria control program (ZAMEP) to target effective interventions to halt local malaria transmission in Zanzibar. Given that the study was conducted during a surge year and in areas known to report relatively high case numbers, the findings offer valuable insights. Specifically, they suggest that outdoor transmission—potentially driven by occupational and behavioural risk factors—may have played a key role in sustaining local malaria transmission during this time.

Occupations with high levels of outdoor activities were associated with increased risk of malaria in urban areas: night watchmen/police, construction workers, and farmers were the salient high-risk occupational groups. Individuals who work in outdoor occupations are not protected from traditional vector control interventions such as insecticide-treated nets (ITNs) or indoor residual spraying (IRS) and are regularly exposed to mosquitoes for extended periods. Additionally, while sex was not assessed as a risk factor in this study, these identified higher-risk occupations are generally more common among men. It was more common to report more than one occupation in rural areas than in urban areas. The connection between occupations with outdoor or manual work and increased risk of malaria has been observed elsewhere, including in Madagascar and Namibia [10, 20]. The risk of malaria infection among farmers in particular has also been observed in Nigeria and South Africa, especially in rice fields that provide ecological conditions favourable for mosquito larva [10, 20, 21].

While the study did not include entomological data collection, assumptions regarding mosquito biting times were based on previous experience at the study sites and unpublished local data. In Zanzibar, the primary malaria vector is *Anopheles arabiensis*, which typically exhibits disproportionately between indoors, 22:00–02:00 h, and outdoors earlier in the evenings, 1800–2100 h [15]. While it is common for watchmen to stay outdoors which directly increases their exposure risk, construction workers in the study areas often live in temporary camps located on-site. These sites frequently include large man-made water storage dams, wells, or unfinished swimming pools, especially where tourist facilities are under construction. The local entomology team has regularly detected *Anopheles* larvae in these water bodies, indicating suitable breeding habitats. Additionally, many workers migrate from mainland Tanzania, where malaria transmission remains higher, raising the likelihood of parasite importation. Combined with factors such as low insecticide-treated net (ITN) usage and night shifts, construction workers may be exposed to mosquito bites during peak vector activity hours.

In both urban and rural areas of Zanzibar, a major behavioural risk factors for malaria is spending time participating in outdoor activities in the evening or early morning, as has been observed in other low transmission settings in Africa and Asia [10, 22]. Night-time activities and taking meals outside at night during the past month were associated with higher odds of malaria in this study. Outdoor activities at night in Zanzibar can include peri-domestic activities including childcare, animal care, cooking, cleaning, and fetching water while outdoor activities away from home can involve social activities, most often during the early evening and night [23].

The linkage between outdoor occupations and outdoor activities with increased risk of exposure to malaria is supported by recent studies in Zanzibar indicating transmission is occurring outdoors due to the mosquito population mainly biting and resting outdoors [19]. In many parts of the malaria endemic world, outdoor biting has been on the rise compared to indoor mosquito-biting behaviour [24–26]. These findings strengthen the evidence of outdoor transmission in these areas of Zanzibar, and further the need to find solutions for occupation and behaviour related malaria exposure [22]. More and better outdoor vector control tools are needed, such as topical repellents, long-sleeved clothing, and larval source management. Information and messaging on outdoor-related malaria risk in rural and urban communities is also required, and an approach to malaria prevention in urban areas specifically, with the global rise in urbanization [27, 28]. However, the transition of information and awareness raising to actual change in behaviour will be challenging as people are outside for many reasons and may perceive malaria risk as low in Zanzibar [1, 22].

Previous studies have found travel to be associated with increased risk of malaria in elimination settings [11, 29, 30]; however, one study in 2015 in Zanzibar found no association between travel within Zanzibar and increased risk of malaria [19]. In this study, results showed that local travel within Zanzibar in the last 3 weeks was a risk factor for malaria among local cases who had not left Zanzibar in both urban and rural districts. Additionally, all three high-risk occupation groups in urban areas reported more recent travel among cases than controls. The higher risk may be due to increased exposure to mosquitoes while traveling, travel to areas of Zanzibar that are particularly high-risk for exposure, staying overnight in informal structures while traveling, or low use of prevention tools while travelling [10, 11, 31]. Previous research in Zanzibar has found that men spend more time away from home than women, indicating they could be at higher risk [23].

Not sleeping under a net has been found to be associated with increased risk of malaria in Zanzibar [19]. In this study, reporting prior night net use was strongly associated with lower odds of malaria in the rural districts, and trended towards being protective in urban districts, though the association did not reach statistical significance in urban districts. Overall, there was relatively low reported net use among the study population: 50% of cases and 58% of controls reported prior night net use. Barriers to ITN use may be heat, perception of low mosquito density during certain seasons, or inability to use ITNs while travelling [23]. A recent study on human behaviour and malaria transmission in Zanzibar (a pre-elimination setting) found that participants reported it was easier to protect themselves from mosquito bites inside than outside because of tools like ITNs that can be used inside [23].

This case–control study has several limitations. First, although it was conducted during a surge year with a marked increase in locally acquired malaria cases—and in areas known for high local transmission, the overall incidence remained relatively low in some districts. This may limit the generalizability of the identified risk factors to other transmission settings or seasons. Second, the small number of cases identified in rural districts reduced the statistical power to detect associations with specific occupations or exposures. The third limitation: generalizability is restricted to treatment-seeking populations who are ill (with malaria or some other cause) and who may differ from the general population in important ways. For example, cases that are not detected at health facilities may be lower density and differ socio-demographically. The test-negative design does have the advantage of avoiding selection bias by ensuring that controls are sampled from the same underlying treatment-seeking populations. Fourth, the use of RDTs for diagnostic testing is a limitation because of the possibility of false negatives, given the inability to detect low density infections, and false positives related to the persistence of HRP2. In the absence of HRP2/3 deletions this misclassification is unlikely to have a major impact on results, given the likelihood that symptomatic malaria generally is accompanied by high parasitaemia. In addition, the study primarily aimed to explore risk factors associated with testing positive for malaria—regardless of species—compared to testing negative; additionally, classification of a case as “locally acquired” versus “imported” was based on the surveillance system’s routine criteria, rather than species identification. Limitations of the data include defining prior-night net use as a dichotomous variable (a continuous measure would have allowed for addressing overnight exposure) and lack of data on indoor residual spraying (IRS) (due to an error in the questionnaire). Lastly, due to the operational nature of the study, it was not feasible to record detailed participant flow information as outlined in the STROBE checklist. Cases were identified and enrolled through the mandatory national surveillance system, with no separate research-led process to document refusals or ineligibility, and no follow-up component. Controls were selected from individuals seeking malaria testing at health facilities and enrolled if they tested negative and matched case profiles by age or sex. While some refusals or ineligible controls may have occurred, this was not systematically recorded, as controls are not typically followed in routine surveillance. Nevertheless, the study reflects a scalable, real-world approach embedded within national systems. It was estimated that over 95% of eligible cases were enrolled, and control recruitment was closely aligned with case enrolment.

There are programmatic implications of these study findings. Delineation of specific high-risk occupations allows for improved malaria surveillance, e.g., adapting case investigation forms to capture specific groups, and allows for targeting interventions that can be effective in these specific settings and to these specific populations. ZAMEP and partners have started initiating these changes: ZAMEP is including the reporting of identified high-risk populations of watchmen and construction workers in the urban districts, and is planning to scale up the monitoring these risk groups. Further information-gathering, such as through qualitative methods, of these priority high-risk populations (watchmen, construction workers, and students) in urban areas with local malaria transmission would build knowledge for targeting and tailoring interventions, such as where and when they congregate, risk perception, access and barriers to diagnosis and treatment, and acceptability of and preferences around potential future interventions.

Moreover, the frequency-matched case–control design used in this study is underutilized in malaria operational research, particularly in low-transmission or pre-elimination settings such as Zanzibar. This approach enabled efficient assessment of associations between exposures and locally acquired malaria while controlling for known confounders like age and sex. This study underscores the value of this design—especially its feasibility, cost-effectiveness, and suitability for identifying risk factors in low-incidence settings—particularly when embedded within routine surveillance activities.

## Conclusions

A detailed understanding of behaviours and occupations that increase risk of malaria is essential to effectively targeting interventions to eliminate malaria in Zanzibar. This study identified three high-risk occupational groups in urban areas of Zanzibar: watchmen/police, construction workers, and farmers, and identified higher risk of malaria in both urban and rural districts among those reporting participating in outdoor activities at night. Travel within Zanzibar was more strongly associated with risk for malaria in urban districts, and prior night net use was more strongly associated with lower risk of malaria in rural districts. Overall, while salient occupational groups at high-risk of malaria were not discernable in the rural districts, other risk factor patterns were similar between the urban and rural districts. More tools are needed to protect individuals from mosquito bites outside the home (for example, spatial repellents, insecticide-treated clothing, or targeted vector control around high-risk worksites may be promising avenues.), and directed interventions to specific high-risk occupational groups can allow for targeting specific reservoirs of local malaria in Zanzibar.

## Data Availability

Data can be made available upon receipt of official requests, and it must ensure participants’ confidentiality and data privacy.
